# 2666. Rates of STI Testing and Diagnoses among Patients with Mpox in New York City, 2022

**DOI:** 10.1093/ofid/ofad500.2277

**Published:** 2023-11-27

**Authors:** Shauna Gunaratne, Simian Huang, Jacob McLean, Clare DeLaurentis, Jason Zucker

**Affiliations:** Columbia University Irving Medical Center, New York, New York; Columbia University Irving Medical Center, New York, New York; New York Presbyterian - Columbia University Irving Medical Center, New York, New York; Columbia University Medical Center, New York, New York; Columbia University Irving Medical Center, New York, New York

## Abstract

**Background:**

The 2022 global mpox outbreak occurred in the context of rising rates of sexually transmitted infections (STIs). We hypothesize that concurrent STI rates were underestimated due to inadequate testing during virtual visits and non-sexual health clinical settings.

**Methods:**

We included data from all patients with confirmed mpox (through non-variola orthopoxviral PCR testing) from June 25 to October 17, 2022 at an urban tertiary academic center in New York. Manual chart review supplemented data extracted from the electronic medical record. We calculated descriptive statistics for HIV, syphilis, genitourinary, rectal, and pharyngeal gonorrhea/chlamydia (GC/CT) screening and diagnosis rates. We evaluated relationships between site-specific symptoms and extragenital testing.

**Results:**

Of 228 individuals in the database, 98% were male at birth, the median age was 34 years. 40% were Hispanic and 27% Black. 50% were living with HIV. Only 41% were tested for other STIs in the two weeks prior to their mpox diagnosis.

At the time of their mpox visit, 62.7% of patients were tested for syphilis, with 45.5% showing evidence of active or previously treated syphilis. 67 persons without known HIV were tested for HIV, resulting in 3 new diagnoses within 2 weeks of mpox diagnosis. 57.5% of patients were screened with a genitourinary GC/CT nucleic acid amplification testing (NAAT), with 2.3% and 3.8% positive for gonorrhea and chlamydia, respectively. 46.5% were screened with a rectal GC/CT NAAT, with 16.0% and 6.6% positive for gonorrhea and chlamydia, respectively. 47.4% of individuals were screened with a pharyngeal GC/CT NAAT, with 7.4% and 0.9% positive for gonorrhea and chlamydia, respectively.

Site specific GC/CT NAATs were obtained from 52.2%, 55%, and 54% of patients with urinary, rectal and pharyngeal signs or symptoms, respectively. Without extragenital testing, 10 of 28 total gonorrhea cases (35.7%) and 3 of 13 total chlamydia cases (23.1%) would have been missed.

**Figure:**
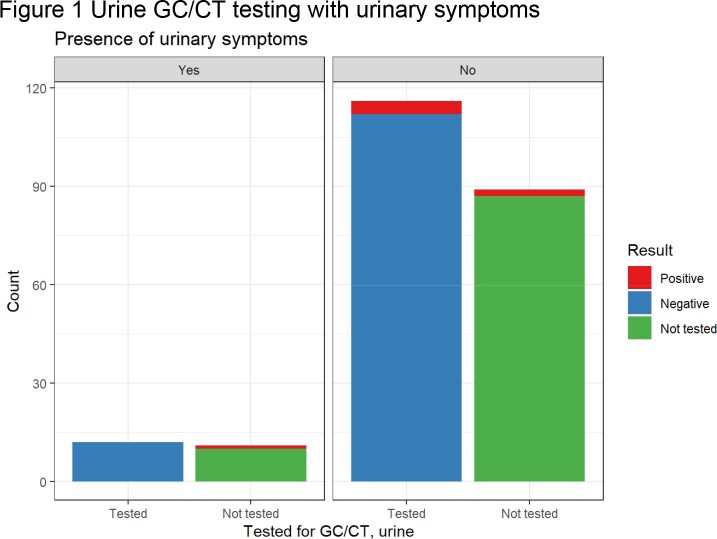

**Figure:**
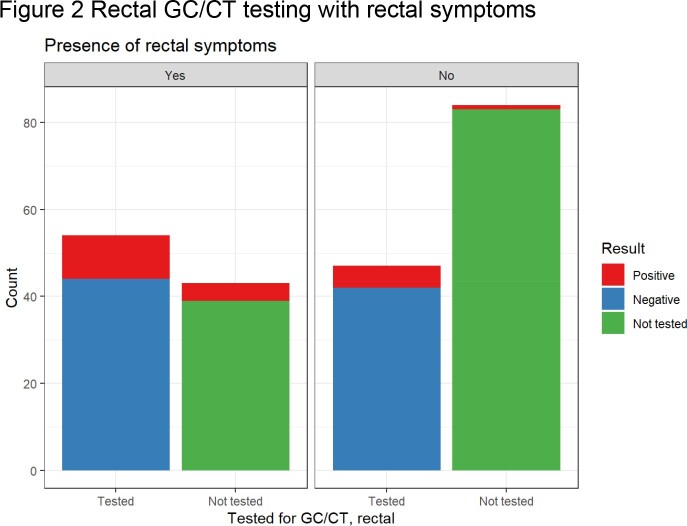

**Figure:**
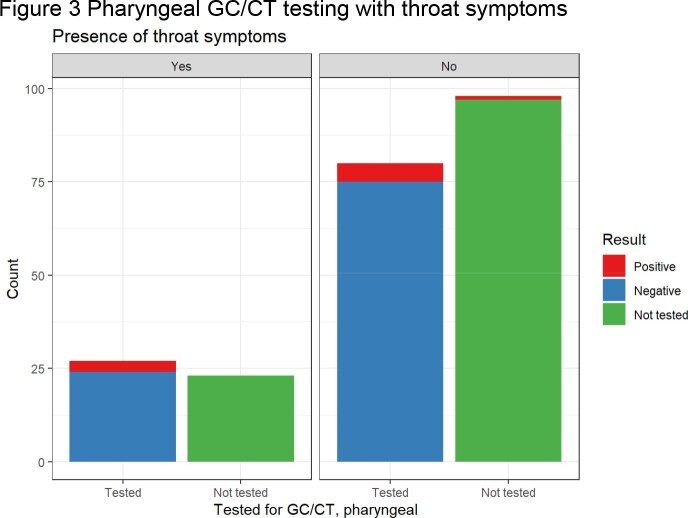

**Conclusion:**

STI testing was insufficient in people with confirmed mpox, likely leading to under diagnosis of STIs. HIV testing was inadequate in at-risk individuals, and patients presenting with site-specific symptoms were too frequently not offered targeted testing.

**Disclosures:**

**All Authors**: No reported disclosures

